# Geographical Influence on Morphometric Variability of Genetically “Pure” *Schistosoma haematobium* Eggs from Sub-Saharan Migrants in Spain

**DOI:** 10.3390/tropicalmed8030144

**Published:** 2023-02-27

**Authors:** Marta Reguera-Gómez, Maria Adela Valero, Patricio Artigas, Alejandra De Elías-Escribano, Maria Cecilia Fantozzi, Maria Pilar Luzón-García, Joaquín Salas-Coronas, Jérôme Boissier, Santiago Mas-Coma, Maria Dolores Bargues

**Affiliations:** 1 Departamento de Parasitología, Facultad de Farmacia, Universidad de Valencia, 46100 Burjassot, Valencia, Spain; 2 Centro de Investigación Biomédica en Red de Enfermedades Infecciosas (CIBERINFEC), Instituto de Salud Carlos III, 28029 Madrid, Madrid, Spain; 3 Unidad de Medicina Tropical, Hospital Universitario Poniente, 04700 El Ejido, Almería, Spain; 4 Host Pathogen Environments Interactions (IHPE) Laboratory, University of Montpellier, CNRS, IFREMER, University of Perpignan Via Domitia, 66860 Perpignan, France

**Keywords:** *Schistosoma haematobium* eggs, morphogenetic analysis, Mali, Mauritania, Senegal, CIAS, measurements, morphotypes, geographical variability

## Abstract

Schistosome eggs play a key role in schistosomiasis diagnosis and research. The aim of this work is to morphogenetically study the eggs of *Schistosoma haematobium* found in sub-Saharan migrants present in Spain, analyzing their morphometric variation in relation to the geographical origin of the parasite (Mali, Mauritania and Senegal). Only eggs considered “pure” *S. haematobium* by genetic characterization (rDNA ITS-2 and mtDNA *cox*1) have been used. A total of 162 eggs obtained from 20 migrants from Mali, Mauritania and Senegal were included in the study. Analyses were made by the Computer Image Analysis System (CIAS). Following a previously standardized methodology, seventeen measurements were carried out on each egg. The morphometric analysis of the three morphotypes detected (round, elongated and spindle) and the biometric variations in relation to the country of origin of the parasite on the egg phenotype were carried out by canonical variate analysis. Mahalanobis distances, when all egg measurements were analyzed, showed differences between: (i) Mali-Mauritania, Mali-Senegal and Mauritania-Senegal in the round morphotype; (ii) Mali-Mauritania and Mauritania-Senegal in the elongated morphotype; and (iii) Mauritania-Senegal in the spindle morphotype. Mahalanobis distances, when spine variables were analyzed, showed differences between Mali-Senegal in the round morphotype. In conclusion, this is the first phenotypic study performed on individually genotyped “pure” *S. haematobium* eggs, allowing the assessment of the intraspecific morphological variations associated with the geographical origin of the schistosome eggs.

## 1. Introduction

Schistosomiasis is caused by blood flukes of the genus *Schistosoma*, which includes species that infect both humans (six species) and animals (twenty species) [1]. Of the 250 million humans infected worldwide, 201.5 million live in Africa, and 110 million of them are affected by *Schistosoma haematobium* [2,3], which causes urogenital schistosomiasis.

On the African continent, considering the species of the genus *Schistosoma* that affect humans, the morphological pattern of the eggs forms two large complexes according to the position of the spine: species whose egg has a lateral spine and species whose egg has a terminal spine. The haematobium group includes eight species (*S. haematobium*, *S. intercalatum*, *S. guineensis*, *S. bovis*, *S. mattheei*, *S. curassoni*, *S. leiperi* and *S. margrebowiei*) that present eggs that have a terminal spine, and freshwater snails of the genus *Bulinus* act as the intermediate host [2,4].

**Figure 1:**
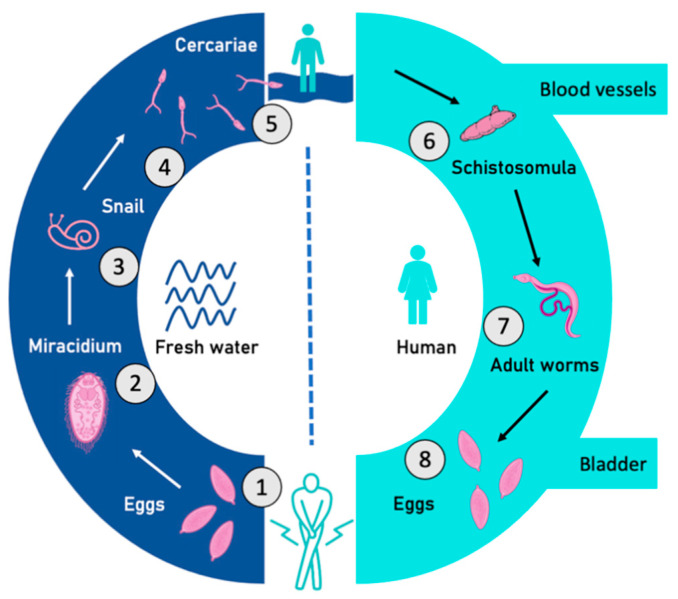
Life cycle of *S. haematobium:* (1) eggs; (2) miracidium; (3) intermediate snail vector; (4–5) cercariae; (6) schistosomula; (7) male and female worms; and (8) release of eggs deposited in veins of the urinary bladder (original from R. Sánchez-Marqués and M.D. Bargues).

Schistosomiasis transmission occurs when miracidia from parasite eggs shed by human urine reach water, where a specific freshwater snail that acts as an intermediate host lives, and a human comes into contact with water contaminated by furcocercariae [5]. Hence, schistosome eggs are also responsible for the occurrence and spread of schistosomiasis. Human activities in infected water promote schistosomiasis transmission. Infection of a human host is part of the highly complex life cycle of the Schistosoma parasite, which is illustrated in Figure 1. The infection by *S. haematobium* (urogenital schistosomiasis) affects the bladder and other pelvic organs. Egg deposition by the adult stage of the parasite in the bladder during infection is associated with hematuria and bladder angioygugenesis [6]. Furthermore, the International Agency for Research on Cancer has categorized urogenital schistosomiasis as a group 1 carcinogen [7], i.e., it is deemed to cause cancer in humans.

In addition to the impact of schistosomiasis in tropical and subtropical areas, infection associated with international travel and migration to Europe has been highlighted [8]. The increased immigration of Africans to Europe has resulted in an increase in the number of patients diagnosed with schistosomiasis in non-endemic areas [9,10].

As a consequence of climate and global change, mosquito-borne infections, snail vectors and snail-borne diseases have become a new challenge for European public health [11,12]. In fact, the presence of autochthonous schistosomiasis in Europe has already been evidenced in countries where the impact of both climate and global change is evident, and the freshwater snail vectors of schistosomiasis of the genera *Bulinus* (*B. truncatus*) and *Planorbarius* (*P. metidjensis*) have been reported [13]. Specifically, human cases have been described on the French island of Corsica [11,14], and it seems that they are no longer restricted to the initial Cavu River focus only [15]. Molecular studies identified both pure *S. haematobium* and hybrids of *S. haematobium* and the cattle parasite *S. bovis* as etiological agents. Moreover, genetically pure *S. bovis* was also reported in a patient from Corsica [2]. The corresponding morphological studies revealed the presence of eggs with terminal spines, which were associated with two morphotypes [16]. In the case of Spain, the number of imported *S. haematobium* cases has increased in recent years, and after an exhaustive clinical and epidemiological study of several cases, autochthonous transmission of urogenital schistosomiasis has been evidenced in Almería (Spain) [17].

In spite of the evident necessity to characterize the etiological agent of the parasitic disease, studies providing a comprehensive genetic and phenotypic characterization of the parasite species have remained scarce. The incorporation of molecular techniques has provided new data and enabled advances in the knowledge of the species of the genus *Schistosoma* [4,18,19,20,21,22]. Even though schistosomes can be identified by species-specific phenotypic characteristics, particularly those associated with the adult worms and their eggs, molecular data have revealed new species distributions, interspecies hybridization and unexpected host associations, all of which highlight the need to incorporate molecular analyses into disease surveillance [22]. Molecular data are particularly relevant in the case of human schistosome eggs excreted in urine to identify pure or hybrid species, as both present a typical terminal spur morphology. Regarding morphologic characterization, a standardized methodology able to provide morphological data complementary to molecular data has been proposed [23]. Furthermore, these new morphometric concepts offer a suitable method to morphologically characterize the phenotype of *Schistosoma* eggs in order to assess the influence of different factors on the shape and size of parasite eggs, such as the geographical origin of the parasite.

The objective of this work is to morphogenetically characterize eggs of *S. haematobium* recovered from sub-Saharan patients living in Spain, analyzing the morphometric variation in relation to the geographical origin of the parasite (Mali, Mauritania and Senegal) and the quantitative characterization of the morphotypes present. To avoid the interference of hybridization, only eggs considered pure *S. haematobium* by genetic characterization (ITS-2 of rDNA and *cox*1 mtDNA) have been included in this study.

## 2. Materials and Methods

### 2.1. Material

**Figure 2:**
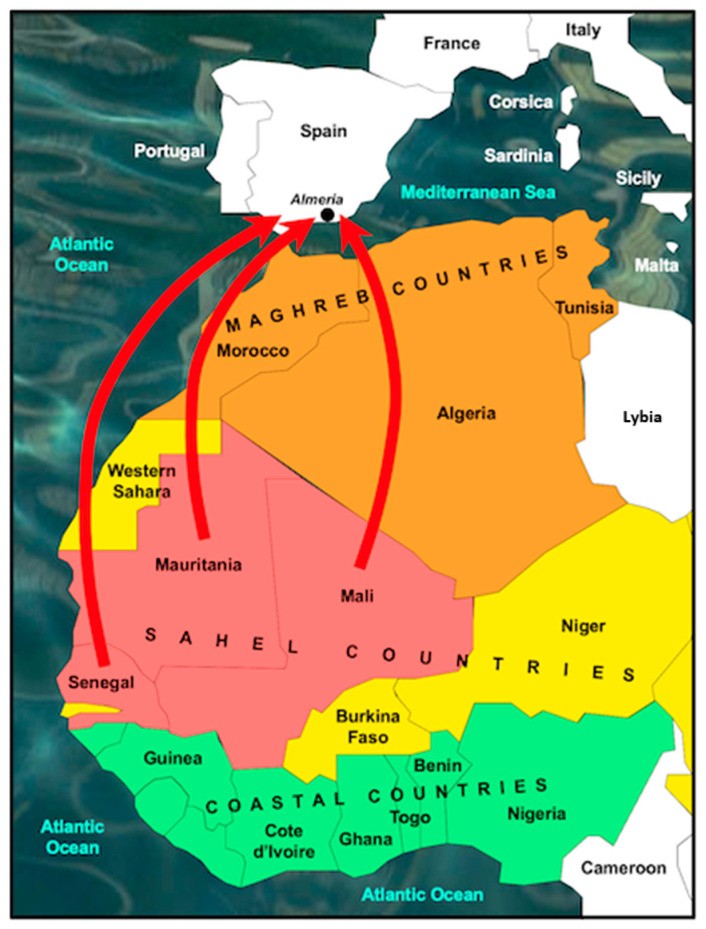
Countries of origin of migrants diagnosed in Spain (Almeria) with urinary schistosomiasis analyzed in this study. Red arrows represent country of origin and final destination.

A total of 162 *S. haematobium* eggs were analyzed (Table 1, Figure 2), recovered from 20 urine samples of migrant men (18–40 years old, average 26.9; 11 from Mali, 4 from Mauritania and 5 from Senegal) living in Spain (from 1 month to 11 years) and diagnosed with urogenital schistosomiasis at the Tropical Medicine Unit of Hospital Universitario Poniente (Almeria, Spain) and considered pure *S. haematobium* by genetic characterization (rDNA ITS-2 and mtDNA *cox*1). Morphological and molecular analyses were performed on the same 162 eggs. Parasite eggs were obtained from urine by using a 40 µm cell strainer (Falcon^®^) and 0.9% NaCl saline solution to wash the sample through the filter. All eggs trapped on the strainer were collected in a Petri dish containing 20 mL of 0.9% saline solution.

As fixation is known to affect the size and shape of helminth eggs, the egg material used in the present study was analyzed without prior fixation, suspended in 0.9% NaCl saline solution, preserved in darkness at 4 °C until required, and digitalized in the shortest possible time, within two weeks after collection [24,25].

### 2.2. Ethical Aspects

Human samples were obtained at the Tropical Medicine Unit of Hospital Universitario Poniente (Almeria, Spain) for diagnostic purposes as part of the standard protocol care for sub-Saharan patients attending such a unit. Anonymized data were collected retrospectively. This study was conducted in accordance with the guidelines of the Declaration of Helsinki and approved by the Institutional Ethics Committee (protocol code PI_19_30 on 25 September 2019). Informed consent was obtained from all subjects involved in the study. At the end of the study, all participants were informed about their parasitological results, and free treatment was offered to those who tested positive for schistosomiasis (single 40 mg/kg dose of praziquantel).

### 2.3. Molecular Analysis of Schistosoma Eggs from Urine Samples

*S. haematobium*-like eggs obtained from the 20 human urine samples were molecularly characterized by a mitochondrial marker (partial cytochrome c oxidase subunit I—*cox*1) and a nuclear marker (complete internal transcribed spacer 2—ITS-2) of the ribosomal DNA. Genomic DNA was extracted individually from each egg using the InstaGeneTM Matrix kit (Bio-Rad Laboratories^®^, Hercules, CA, USA) following the manufacturer’s instructions and stored at −20 °C until use.

For mitochondrial *cox*1 profiling, an RD-PCR of each of the 162 eggs was performed using one universal forward (5′-TTTTTTGGTCATCCTGAGGTGTAT-3′) and three reverse species-specific primers (5′-CACAGGATCAGACAAACGAGTACC-3′, 5′-TGCAGATAAAGCCACCCCTGTG-3′ and 5′-TGATAATCAATGACCCTGCAATAA-3′) [26,27,28] to amplify a specific mtDNA *cox*1 region (differing in length) for *S. bovis* (306 bp), *S. mansoni* (375 bp) and *S. haematobium* (543 bp). The PCR reactions were carried out in a final volume of 12.6 μL, comprising 8 μL of DNA template, 2.5 μL of 5X Colorless GoTaq Flexi Buffer, 0.75 μL of MgCl_2_ (25 mM), 0.25 μL of dNTP (10 mM), 1 μL of primer mix (1 μM), 0.1 μL of GoTaq G2 Hot Start Polymerase (Promega, Madison, WI, USA) and using PCR conditions as previously described [23]. The amplified products were separated electrophoretically on a 2.5% agarose gel containing GelRed (Biotium, San Francisco, CA, USA).

The information provided by the maternally inherited mtDNA profiling was complemented with the partial sequence of the 5.8S rRNA gene and the complete ITS-2 of the rDNA. PCR amplification and sequencing were performed independently on each of the 162 eggs using primers 3S and A28S [23,29,30,31], designed in the flaking regions of the 5.8S and 28S rRNA genes. PCR conditions were an activation step of 4 min at 94 °C, followed by 32 cycles of 55 secs at 94 °C, 1 min at 55–62 °C and 1.30 min at 72 °C with a final extension of 5 min at 72 °C followed by a final cooling at 4 °C. PCR amplifications were performed in a Veriti 96-Well Thermal Cycler (Applied Biosystems, Thermo Fisher Scientific, Waltham, MA, USA). Purified PCR products were resuspended in 50 μL of 10 mM TE buffer (pH 7.6). The final DNA concentration (in μg/mL) and absorbance at 260/280 nm were determined in an Eppendorf BioPhotometer (Hamburg, Germany). Sequencing was performed on both strands by the dideoxy chain-termination method, using the Taq dye-terminator chemistry kit on an Applied Biosystems 3730xl DNA Analyzer (Applied Biosystems, Foster City, CA, USA).

The software Sequencher version 5.4.6 (Gene Codes Co., Ann Arbor, MI, USA) was used to edit and assemble the sequences, and ClustalW to align them by means of default parameters in MEGA X [32]. A careful inspection of all nucleotide positions in the electropherograms (ABI format) allowing the detection of sequence polymorphisms between *S. haematobium* and *S. bovis*, was performed to identify possible heterogeneity, as previously described [4,33,34]. Homologies were performed using the BLASTN program from the National Center for Biotechnology Information website (http://www.ncbi.nlm.nih.gov/BLAST, accessed on 29 September 2022).

### 2.4. Schistosome Egg Digitalization and Measurements

Schistosome egg measurements were performed on 162 samples (Table 1). Each egg was individualized in a 0.9% NaCl drop on a slide and photographed using a microscope (Leica DMR 72-89663) with 400 magnifications and a high-resolution camera (Leica DFC450C) controlled by the LAS LEICA software version 4.3 (Leica Microsystems, Heerbrugg, Switzerland).

All pictures were taken without a coverslip. No pressure was applied. In addition, the lack of a coverslip allowed the recovery of individual eggs and their preservation in EtOH at 70 °C for posterior molecular characterization. For morphologic characterization, the Image Pro Plus version 5.1 software (Media Cybernetics Inc., Silver Spring, MD, USA) was used.

**Figure 3:**
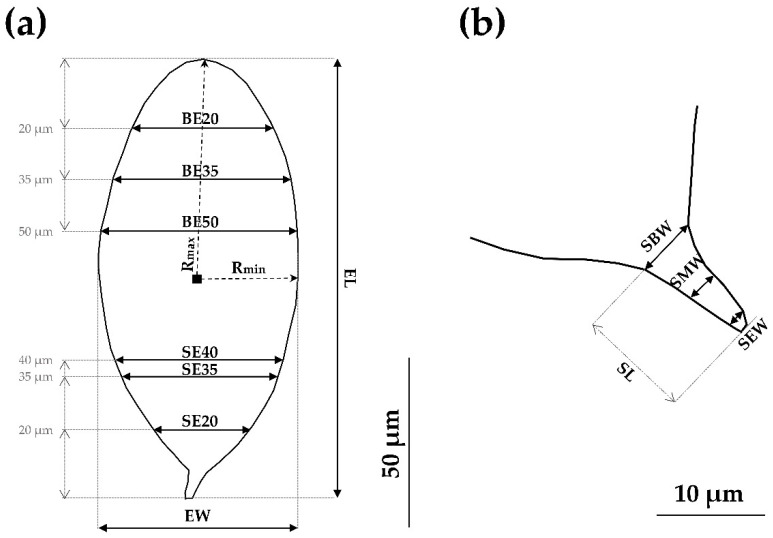
(**a**) Standardized measurements designed for the morphometric phenotyping of *S. haematobium* eggs. (**b**) Detail of standardized spine measurements.

Following the already standardized methodology [23,25,35], the 17 non-redundant measurements used in this study were (Figure 3): Egg Area (EA), Egg Perimeter (EP), Radius max (Rmax), Radius min (Rmin), Egg Length (EL), Egg Width (EW), Egg Roundness (ER), width at 20 µm from Blunt End (BE20), width at 35 µm from Blunt End (BE35), width at 50 µm from Blunt End (BE50), width at 20 µm from Spine End (SE20), width at 35 µm from Spine End (SE35), width at 40 µm from Spine End (SE40), Spine Length (SL), Spine Base Width (SBW), Spine Medium Width (SMW) and Spine End Width (SEW). Furthermore, the following ratios were calculated: Length/Width Ratio (LWR), Egg Shape Ratio (ESR) and Elongation Ratio (ERatio), where LWR = EL/EW, ESR = EL/(BE35 × ER) and ERatio = Rmax/Rmin.

### 2.5. Statistical Data and Analyses

Statistical analyses were conducted using R version 4.0.0 software (The R Project for Statistical Computing; http://www.rproject.org, accessed on 20 October 2022). Generalized Linear Mixed Models (GLMM) were calculated. All models included patient identification as a random intercept to account for the lack of independence of observations (host sample size effects). Measurements were used as a response variable, and host sample size effect and geographical location were used as independent variables. The ‘glmer’ function of the lme4 package was used. The relevance of the terms was evaluated with the second-order Akaike Information Criterion (AICc) to account for small sample sizes [36,37]. When the inclusion of each term did not reduce AICc values by 2 or more units (ΔAICc < 2), it was dropped from the model. Results were considered statistically significant when *p* < 0.05.

A multivariate analysis was used on the biometric data obtained [38]. Geometrical morphometrics was used to quantify the morphological differences observed, which includes validated methods for analyzing variation in organismal shape [39,40].

Size-free canonical discriminant analysis was used on the covariance matrix of measurements transformed into logarithms to assess morphometric variation among samples [41]. 

The resulting “allometry-free” (effect of size on morphological variation) or size-free variables were submitted to canonical variate analysis (CVA), and Mahalanobis distances were derived [42]. In the present analysis, the degree of shape divergence between *S. haematobium* egg populations was assessed through pairwise Mahalanobis distances [43,44,45]. CVA and Mahalanobis distances were performed using both the CLIC package and the available online XYOM software (https://xyom.io/ accessed on 14 February 2023) and tested by non-parametric permutation tests with 1000 permutations each. Values were considered statistically significant when *p* < 0.05 [23,46,47,48]. Nevertheless, to avoid matrix singularities, ratios were discarded because of their overlap with other measurements, and only the 17 above-mentioned non-redundant measurements were used.

## 3. Results

A total of 162 eggs, including 66 (40.7%) from Mali, 55 (34.0%) from Mauritania and 41 (25.3%) from Senegal, were morphologically phenotyped and genetically characterized (Table 1). Certainty about the country of infection can be assumed from the patient’s anamnesis. Patients whose epidemiological records were not accurate or ambiguous (not certain about the place of infection) were not included in the study.

### 3.1. Genetic Characterization

Both molecular markers (rDNA and mtDNA) were coincident, allowing us to confirm that all 162 schistosome terminal-spined eggs from urine samples analyzed belonged to the genetic profile of pure *S. haematobium*.

#### 3.1.1. Cox1 Rapid Diagnostic PCR

**Figure 4:**
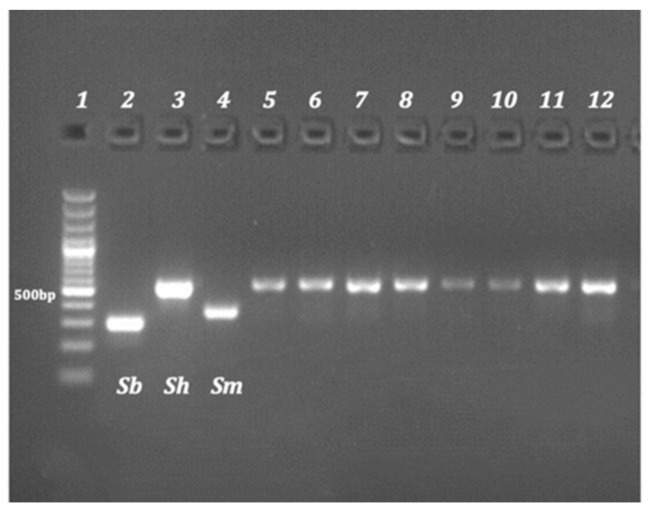
Mitochondrial *cox*-1 profile by RD-PCR. Lane 1: DNA ladder (100–3000 bp); lane 2: *S. bovis* (306 bp); lane 3: *S. haematobium* (543 bp); and lane 4: *S. mansoni* (375 bp). Each of the following lanes (5–12) corresponds to a separate multiplex PCR containing DNA extracted from one single egg, where lanes 5–8 match with the eggs of a patient from Mali, 9–10 from Senegal and 11–12 from Mauritania.

The *cox*1 RD-PCR results obtained were identical for all 162 eggs individually analyzed and correspond to the *S. haematobium cox*1 genetic profile. No eggs/miracidium provided a profile of *S. bovis* or *S. mansoni* (Figure 4).

#### 3.1.2. ITS rDNA Sequence Analysis

The rDNA sequences demonstrate that the *S. haematobium* profile was “pure” in all the eggs analyzed. No hybrid profiles were detected in the chromatograms. Among the 162 sequences obtained of 452 bp, including the partial 5.8S rRNA (138 bp) and the complete second Internal Transcribed Spacer (ITS-2) (314 bp), two pure *S. haematobium* haplotypes were obtained (Sh-1 and Sh-2). After a comparative analysis with other *S. haematobium* sequences from other countries (Africa and Europe), *S. bovis*, and *S.haematobium x S. bovis*, available in GenBank (Table 2), the alignment carried out allowed us to observe that Sh-1 showed sequence identity (100%) with *S. haematobium* (consensus sequence of GenBank Acc. Nos. GU257398, JQ397400–JQ397414, FJ588861, MT580953, MT884914 and MT158873) and Sh-2 was 100% identical to that previously described in the Ivory Coast (MG554667) and in Corsica, France (MW130296). This haplotype (Sh-2) contains a heterozygotic signal (C/T) in position 60 of the 5.8S-ITS-2 alignment and did not correspond to any discriminative position among other *Schistosoma* species with terminal-spined eggs. The haplotypes Sh-1 and Sh-2 were present in 41.36% (67/162) and 58.64% (95/162) of the total samples, respectively, and in patients from Mali, Mauritania and Senegal, with Sh-2 being more abundant than Sh-1 in all three countries.

### 3.2. Phenotypic Characterization

#### 3.2.1. Morphotypes of *S. haematobium* Eggs

**Figure 5:**
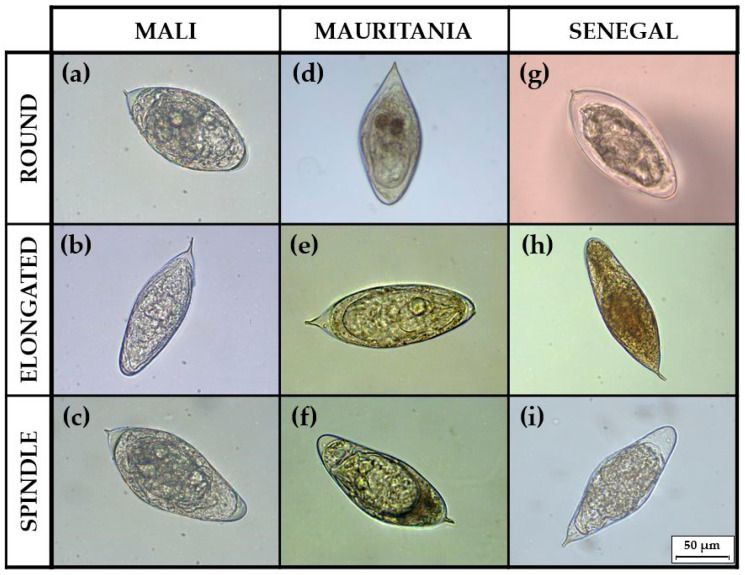
Representative egg samples of the different morphotypes from each country: (**a**) round, (**b**) elongated, and (**c**) spindle eggs from Mali; (**d**) round, (**e**) elongated, and (**f**) spindle eggs from Mauritania; (**g**) round, (**h**) elongated, and (**i**) spindle eggs from Senegal.

Only eggs genotyped as pure were used in the study. In order to establish a phenotypic characterization, three morphotype classes were described based on the results of the Egg Shape Ratio (ESR): (a) morphotype round (ESR < 2.00); (b) morphotype elongated (2.01 < ESR < 2.29); and (c) morphotype spindle (ESR > 2.30). Representative images of the three different morphotypes of *S. haematobium* eggs from Mali, Mauritania and Senegal are shown in Figure 5. In the 162 eggs analyzed, 55 (34.0%) presented a round morphotype, 62 (38.3%) were elongated, and 45 (27.7%) presented a spindle morphotype.

#### 3.2.2. Influence of the Geographical Origin of the Parasite on the Size of *S. haematobium* Eggs

The size of *S. haematobium* eggs obtained from human samples from Mali, Mauritania and Senegal is shown in Table 3. Overall, *S. haematobium* eggs from Senegal showed the greatest length, area, perimeter and radius. Eggs from Mali have greater roundness and Spine Medium Width (SMW), while Mauritanian eggs are characterized by the smallest radius and width. The comparison of measurements of *S. haematobium* eggs coming from these three countries is shown in Table 4, presenting differences in both measurements and ratios between eggs from Mali and Senegal.

**Figure 6:**
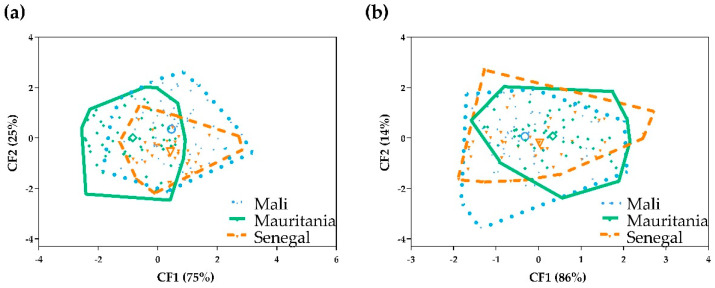
Factor map corresponding to *Schistosoma haematobium* eggs shed by humans from Mali, Mauritania and Senegal: (**a**) all measurements analyzed; (**b**) spine measurements analyzed. Each group is represented by its perimeter. Dotted line: Mali; solid line: Mauritania; dashed line: Senegal. Circle: Mali centroid; square: Mauritania centroid; triangle: Senegal centroid.

Furthermore, the study of the influence of the geographical location on the size of *S. haematobium* eggs was carried out by CVA. When analyzing all measurements, the corresponding factor maps (Figure 6a) illustrate global size differences, and five areas can be distinguished: one corresponds to eggs from Mali; one consists only of samples from Mauritania; and the other three contain eggs from Mali and Senegal, Mali and Mauritania, or an overlap between eggs coming from Mali, Mauritania and Senegal. When spine measurements are analyzed, the corresponding factor maps (Figure 6b) illustrate an overlap between the eggs from Mali, Mauritania and Senegal.

Mahalanobis distances between the three *S. haematobium* populations (Mali, Mauritania and Senegal) (Table 5) show statistically significant differences between Mali, Mauritania and Senegal when all measurements are taken into account and between Mali and Mauritania and Mali and Senegal when only spine measurements are considered.

#### 3.2.3. Morphotypes and Biometric Variations in Relation to the Geographical Origin of the Parasite

The percentages of each morphotype of genetically pure eggs by country of origin are detailed in Table 6. The chi-square test did not show a non-random association (*p* = 0.125) between country and morphotype, i.e., morphotypes are distributed equally among countries.

**Figure 7:**
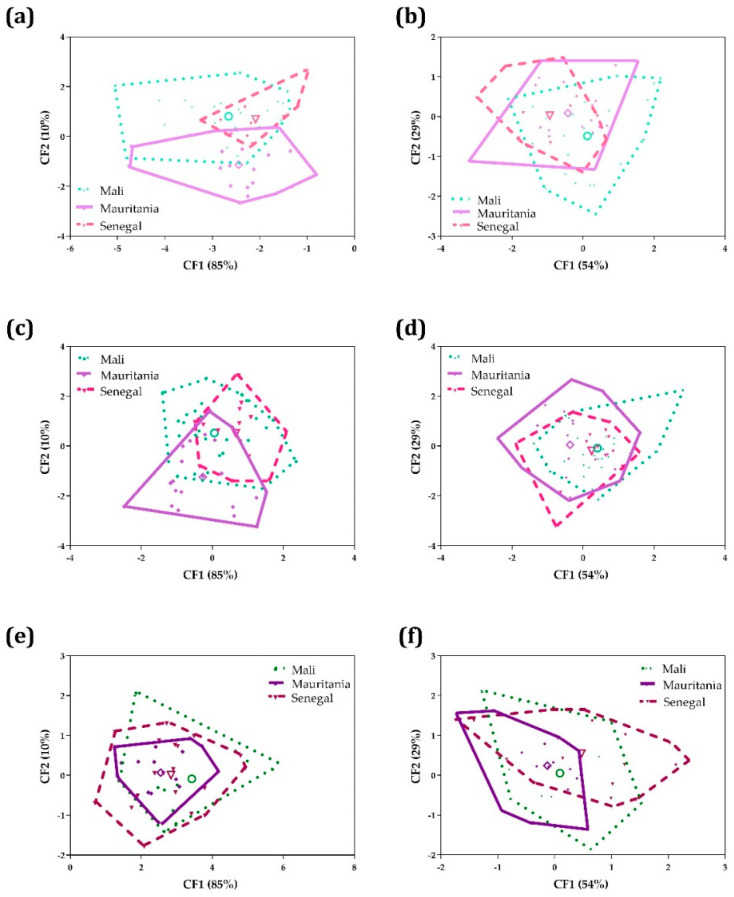
Factor map corresponding to *S. haematobium* eggs shed by individuals from Mali, Mauritania and Senegal: (**a**) analysis of all measurements of the round phenotype; (**b**) analysis of spine measurements of the round phenotype; (**c**) analysis of all measurements of the elongated phenotype; (**d**) analysis of spine measurements of the elongated phenotype; (**e**) analysis of all measurements of the spindle phenotype; and (**f**) analysis of spine measurements of the spindle phenotype. Each group is represented by its perimeter. In order to facilitate the comparison between countries, each morphotype is displayed separately, but graphs (**a**,**c**,**e**) and (**b**,**d**,**f**) come from the same CVA analysis, respectively. Dotted line: Mali; solid line: Mauritania; dashed line: Senegal. Circle: Mali centroid; square: Mauritania centroid; triangle: Senegal centroid.

The morphometric analysis of the three morphotypes detected (round, elongated and spindle) and the biometric variations in relation to the country of origin of the parasite on the egg phenotype were carried out by CVA. First, all variables of the three morphotypes were analyzed, and factor maps were plotted separately for round (Figure 7a), elongated (Figure 7c) and spindle (Figure 7e) morphotypes. Likewise, spine variables of the three morphotypes were analyzed jointly, and factor maps were plotted separately for round (Figure 7b), elongated (Figure 7d) and spindle (Figure 7f) morphotypes. The resulting factor maps (Figure 7) clearly illustrate global size differences in the egg populations analyzed, including: (a) a larger global size in eggs from Mauritania but a larger spine size in eggs from Mali in the round morphotypes (Figure 7a,b); (b) a larger global size in eggs from Mali and a larger spine size in eggs from Mali in the elongated morphotypes (Figure 7c,d); and (c) a larger global size in eggs from Mali and a larger spine size in eggs from Senegal in the spindle eggs (Figure 7e,f).

Mahalanobis distances (Table 7) between the three morphotypes detected (round, elongated and spindle) in *S. haematobium* eggs and the three countries of origin of the parasite populations (Mali, Mauritania and Senegal) showed statistically significant differences, when all measurements were analyzed (Table 7a), between: (i) Mali-Mauritania, Mali-Senegal and Mauritania-Senegal in the round morphotype; (ii) Mali-Mauritania and Mauritania-Senegal in the elongated morphotype; and (iii) Mauritania-Senegal in the spindle morphotype. Statistically significant differences were found in spine variables (Table 7b) between Mali-Senegal in the round morphotype.

## 4. Discussion

In general, geographical region as well as intermediate snail and definitive mammalian host associations are used as diagnostic criteria during a trematodiasis outbreak [49]. However, trematode eggs are specific morphological structures that play a key role in the diagnosis of the disease [25]. In this sense, schistosomes can be identified by species-specific phenotypic traits associated with the parasite eggs. The greater morphological complexity of schistosome eggs when compared to other trematode species is noteworthy and might give rise to complex epidemiological controversies. The haematobium complex presents a challenge when including species of both medical (*S. haematobium*, *S. intercalatum* and *S. guineensis*) and veterinary (*S. bovis*, *S. mattheei* and *S. curassoni*) [50] importance. The identification of *Schistosoma* egg species is yet even more problematic due to the presence of intraspecific morphometric variation and potential natural hybridizations, as has already been reported both in nature and experimentally [1,2,51,52,53,54,55,56].

Given the great complexity of the shapes of the eggs of *Schistosoma* species, there are a very limited number of studies focused on the intraspecific variability of their shape and size. In terms of the species that produces eggs with lateral spines, morphometric studies of *S. mansoni* eggs on the island of Guadalupe revealed intrapopulation variability [57]. In the group of schistosomes whose eggs have terminal spines, the inter-specific [23,58,59] and intra-specific variability [60,61,62] have been the subject of different studies in which biometric markers have been used to facilitate identification. Reguera-Gomez et al. [23] proposed the standardization of seventeen measurements applied to *Schistosoma* egg species with lateral and terminal spines. Surprisingly, until then, the distinctive morphology of *Schistosoma* eggs had mainly been characterized only by their length, width and length/width ratio. However, it is worth mentioning that studies performed on *S. intercalatum* eggs showed that the length/width ratio is highly variable [63]. Furthermore, these classical morphometric measurements do not allow for the discernment of the “spindle” shape displayed by some morphological phenotypes.

Previous studies characterizing *S. haematobium* eggs showed great variability in their phenotype and measurements, demonstrating the necessity of new studies focused on the influence of distinct factors that may potentially be involved, such as the geographical origin of the parasites. In the present work, the intraspecific variability associated with the country of origin of the parasite is characterized, as well as the phenotypes present in each country, using the previously proposed methodology [23]. According to previous descriptions of *S. haematobium* egg measurements, EL varied from 83 µm to 170 µm in eggs recovered from human urine samples [23,34,59,64]. The average length of the *S. haematobium* eggs analyzed herein (132.30 µm, range: 106.29–163.61 µm, for eggs coming from Mali; 137.50 µm, range: 116.30–164.36 µm, for eggs from Mauritania and 148.70 µm, range: 128.10–164.80 µm, for eggs from Senegal) falls within this range, although our minimum record is larger. The maximum width (70.11 µm) detected in the *S. haematobium* eggs from Mali analyzed in the present study is also significantly smaller than that previously described (95 µm) [34], probably due to the use of the coverslip in similar studies.

In 2013, schistosomiasis was reported from southern Europe (Corsica, France), and endemic infections were repeatedly identified in 2015 and 2016 [65]. Moné et al. [16] genetically identified the presence of both “pure” *S. haematobium* and hybrid crosses between *S. haematobium* and *S. bovis* in eggs isolated from infected patients. Regarding the morphological characterization, the eggs were mounted individually in a 9‰ NaCl saline solution under coverslips on glass slides. The average of 15 eggs was 106.5 µm in length and 42.8 µm in width, which is smaller than the respective averages of the eggs found in Mali, Mauritania and Senegal described in the present study. Two egg morphotypes, with significantly different proportions, were identified in the above-mentioned 15 eggs shed by patients from Corsica: (a) the typical round to oval one, shared by 26.7% of the eggs; and (b) a more elongated and lozengical one, common in the majority of the eggs (73.3%) [16]. Unfortunately, morphological and genetic characterization were not performed on the same eggs, and the morphotype and genotype of hybrid eggs have so far not been associated.

The present work allows for the classification of eggs according to their shape (standardized by ESR values) into three morphotypes: round (ESR < 2.00), elongated (2.01 < ESR < 2.29) and spindle (ESR > 2.30). This new morphological biomarker makes it possible to avoid subjective classification into one or another morphological phenotype. In this study, genetically “pure” *S. haematobium* eggs coming from Mali, Mauritania and Senegal have been classified into these three morphotypes (round, elongated and spindle) and compared between countries. The results suggest that *S. haematobium* eggs show great shape variability within the three populations, and, unexpectedly, round or elongated shapes are not always the most common phenotypes of genetically “pure” *S. haematobium* eggs, as previously believed.

As suggested above, the characterization of the size and shape of *Schistosoma* spp. eggs could be more accurately determined when using both additional measurements and new morphometric techniques [26,66]. The morphometric characterization of populations of *S. haematobium* eggs derived from urine samples of patients from Mali, Mauritania and Senegal was carried out using this methodology, taking into account standardized measurements, geographical location influence, and its correlation with morphotypes.

It is well known that the variation of the morphological phenotype present in the populations of free-living species often becomes evident when they come from different geographic areas or when they have undergone pronounced changes in their environment, which has been the subject of a great number of studies since the Neo-Darwinian synthesis [67]. In recent years, geometric morphometrics has been shown to be a useful tool for the characterization and differentiation of adults or eggs of helminths such as those of the genera *Fasciola* [25,31,68,69,70], *Trichuris* [44,48] or *Calodium* [45]. In the case of *Fasciola* species, the presence of adult and egg morphological variability associated with geographic location has been described in multiple studies [25,31,41,43,70,71,72].

Quantitative morphological variation can inform about genetic variation as well as external influences [73]. In the case of endoparasites, their macrohabitat (external environment according to geographical area) and microhabitat (affected organ inside the host) can be distinguished [70]. Studies carried out on *S. intercalatum* described two morphological patterns when eggs from Cameroon and the former Zaire were compared [74]. This study evinced the presence or absence of a shoulder at the base of the spine. The shoulder was well marked in the case of the strain from Cameroon but absent in the case of the strain from the former Zaire. This feature had already been evidenced by Frandsen [75], as well as an asymmetry of the egg from its major axis, which was more frequent in the strain from the former Zaire than in the strain isolated from Cameroon. Furthermore, the eggs of the Cameroon strain were larger and narrower than those of the strain from the former Zaire. Egg size variability has also been described within two experimental populations of *S. haematobium* from Algeria, using the same intermediate snail host, *Bulinus truncatus*, but originating from two distinct ecological areas. The analysis showed differences in the growth rate of adult worms, the size and shape of the eggs, the chronobiology of cercarial emergence and the compatibility with the intermediate host, indicating intraspecific polymorphism [70,76].

On the other hand, it is noteworthy that the sexual reproduction of schistosomes in their definitive hosts is a characteristic that allows the rearrangement and perpetuation of the genotypic diversity of the parasite [77]. In recent years, numerous studies have reported this phenomenon, analyzing different parasite developmental stages. For example, based on the microsatellite analysis of individual miracidia from human *S. haematobium* eggs, high levels of genetic diversity have been detected at the country level but not at the regional level across six sub-Saharan African countries [78]. Furthermore, significant variance in genetic diversity and differentiation among populations of *S. haematobium* in Africa, using DNA markers for individual adult worms of *S. haematobium* from laboratory animals [79] has been described. Specifically, significant differences in the genetic variation of *S. haematobium* populations from Mali and Nigeria have also been found [80]. However, in general, the first major characteristic of *S. haematobium* is that this species displays very low genetic diversity, i.e., only two major clades have been identified based on their mitochondrial haplotypes across its range of distribution in Africa [81]. Moreover, microsatellites do not appear to be useful to distinguish structured populations [82]. The difference we found between the morphotypes of *S. haematobium* in each country is, therefore, surprising. Hence, the analysis of egg morphotypes is all the more interesting in this context, where genetics is limited. All in all, our results on the *S. haematobium* egg phenotype suggest an absence or insufficiency of genetic exchange between neighboring countries, which in the long term would lead to country-specific morphotypes.

This complex genetic diversity makes egg characterization very challenging, as thus far there is no *S. haematobium* egg population characterized as a standard for phenotypic comparisons in future studies to analyze the impact of hybridization on egg morphology. This is the first phenotypic study performed on individually genotyped “pure” *S. haematobium* eggs, based on both ribosomal and mitochondrial markers, thus allowing for a true investigation of the intraspecific morphological variations associated with a given variable, in this case, the geographical origin of the schistosomes. We have confirmed that morphological variations of “pure” *S. haematobium* eggs differ according to the parasite’s geographical origin (Mali, Mauritania and Senegal) and agree with previous studies concerning the size and shape variability of *Schistosoma* eggs. Furthermore, the morphometric results described herein agree with previous findings describing phenotypic variations of *S. haematobium* egg populations in Africa at the country level [26].

As with any other microscopy-based method, this study has its limitations. Eggs are three-dimensional structures with bilateral symmetry. The characterization performed here is based on a 2D image that could vary depending on the position of the eggs placed on the slide.

Finally, urinary schistosomiasis caused by *S. haematobium* shows significant phenotypic differences in epidemiology, clinical manifestations and transmission [83]. The morphological phenotyping of the parasite eggs can provide complementary information to that already obtained by existing tools to further understand the scope of these differences and their subsequent influence on the impact of the pathogenesis, treatment, and control of schistosomiasis worldwide.

## Figures and Tables

**Table 1:** Number of eggs phenotyped and genotyped for each of the 20 patients (P) included in this study, according to their country of origin.

Country	Patient Code	No. Eggs Morphogenetically Analyzed
MALI	P-1	1
	P-6	4
	P-7	5
	P-9	9
	P-11	5
	P-12	8
	P-13	7
	P-14	5
	P-17	4
	P-22	12
	P-23	6
	Total	66
MAURITANIA	P-15	3
	P-30	9
	P-34	23
	P-49	20
	Total	55
SENEGAL	P-4	9
	P-18	2
	P-19	7
	P-20	9
	P-21	14
	Total	41
Total		162

**Table 2:** Variable positions in the 5.8S and ITS-2 sequence alignment of Sh-1 and Sh-2 haplotypes of *S. haematobium* obtained in this study and other *S. haematobium*, *S. bovis* and *S. haematobium x S. bovis* sequences from GenBank. The numbers (to be read vertically) refer to variable positions obtained in the alignment made with MEGA X; . = identical; - = not sequenced. Heterozygotic positions are represented with the corresponding symbol of the IUPAC code for an incomplete nucleic acid specification.

Species	Country	Variable Positions 5.8S + ITS-2 Alignment	
		5.8S	ITS-2
		5 9	2234444 2830555 8333012
*S. haematobium* *S. haematobium* Sh-1 *S. haematobium* Sh-2 *S. haematobium* *S. bovis* *S. haematobium x S. bovis*	African countries ^1^ Mali, Mauritania and Senegal Mali, Mauritania and Senegal Ivory Coast and France Senegal Benin	C . Y Y . Y	GCGCCCC ....... ....... ....... ATAT--- RYRY---

^1^ Tanzania (GenBank Acc. No. GU257398); Senegal, Mali, Gambia, Madagascar, Mauritius, South Africa, Zambia, Malawi, Tanzania, Sudan, Egypt, Cameroon, Nigeria, Guinea Bissau, and Liberia (JQ397400–JQ397414); Senegal (FJ588861 and MT580953); Zimbabwe (MT884914); and Benin (MT158873).

**Table 3:** Measurements of *S. haematobium* eggs grouped by country of origin. Mean ± SD (Min–Max).

	Mali (N = 66)	Mauritania (N = 55)	Senegal (N = 41)
Egg Area (EA) (µm^2^)	5060 ± 922.37 (2921.43–7391)	5175 ± 875.97 (2933–7231.93)	5542 ± 704.62 (4393–7851)
Radius maximum (ERmax) (µm)	70.44 ± 6.95 (55.85–84.82)	72.75 ± 5.83 (63.00–91.12)	77.94 ± 4.68 (67.54–86.51)
Radius minimum (Rmin) (µm)	27.25 ± 2.93 (19.77–34.45)	26.86 ± 3.25 (19.41–33.57)	27.00 ± 2.53 (22.87–33.77)
Egg Perimeter (EP) (µm)	299.90 ± 29.09 (243.20–356.50)	307.90 ± 22.99 (251.7–363.28)	329.10 ± 17.98 (287.70–372.00)
Egg Length (EL) (µm)	132.30 ± 14.31 (106.29–163.61)	137.50 ± 10.29 (116.30–164.36)	148.7 ± 8.46 (128.10–164.00)
Egg Width (EW) (µm)	56.05 ± 5.80 (40.27–70.11)	55.38 ± 6.37 (39.06–67.93)	56.04 ± 4.77 (46.29–69.61)
Egg Roundness (ER)	1.43 ± 0.12 (1.22–1.75)	1.48 ± 0.10 (1.31–1.81)	1.57 ± 0.10 (1.35–1.82)
Width at 20 µm from Blunt End (BE20) (µm)	36.33 ± 4.48 (25.77–46.25)	34.13 ± 3.37 (24.45–41.19)	31.34 ± 2.89 (23.92–39.06)
Width at 35 µm from Blunt End (BE35) (µm)	46.52 ± 5.73 (33.75–58.47)	43.67 ± 4.48 (33.22–54.22)	39.97 ± 4.22 (31.88–52.09)
Width at 50 µm from Blunt End (BE50) (µm)	53.48 ± 6.06 (39.34–66.16)	51.41 ± 5.42 (38.53–64.56)	47.71 ± 5.20 (39.06–63.50)
Width at 20 µm from Spine End (SE20) (µm)	25.18 ± 5.20 (14.62–38.25)	23.82 ± 5.10 (15.15–39.34)	22.52 ± 4.07 (15.15–31.09)
Width at 35 µm from Spine End (SE35) (µm)	40.59 ± 5.89 (23.12–54.75)	38.15 ± 5.32 (27.64–52.34)	37.01 ± 4.80 (29.50–47.56)
Width at 40 µm from Spine End (SE40) (µm)	44.69 ± 5.88 (27.37–57.94)	42.17 ± 5.41 (31.34–54.47)	41.04 ± 4.94 (33.22–53.16)
Spine Length (SL) (µm)	7.71 ± 2.80 (3.24–15.94)	7.45 ± 2.27 (3.18–12.53)	8.62 ± 2.44 (3.98–16.15)
Spine Base Width (SBW) (µm)	4.87 ± 1.56 (2.39–10.10)	4.64 ± 1.31 (2.12–7.86)	4.73 ± 1.14 (2.65–7.44)
Spine Medium Width (SMW) (µm)	2.79 ± 0.64 (1.41–4.38)	2.66 ± 0.69 (1.18–4.25)	2.54 ± 0.57 (1.35–3.98)
Spine End Width (SEW) (µm)	1.73 ± 0.56 (0.59–3.18)	1.61 ± 0.43 (0.75–2.45)	1.61 ± 0.43 (1.06–2.45)
Length/Width Ratio (LWR)	2.37 ± 0.26 (1.91–3.06)	2.50 ± 0.24 (2.08–3.30)	2.66 ± 0.22 (2.12–3.25)
Egg Shape Ratio (ESR)	2.00 ± 0.26 (1.64–2.75)	2.13 ± 0.09 (1.90–2.29)	2.38 ± 0.17 (2.01–2.93)
Elongation Ratio (ERatio)	2.60 ± 0.30 (2.05–3.32)	2.73 ± 0.30 (2.21–3.72)	2.90 ± 0.29 (2.27–3.51)

**Table 4:** Significantly different measurements between *S. haematobium* eggs compared by the geographical origin of the parasite (Mali, Mauritania and Senegal) by GLMM analysis (*p* < 0.05). All models were made, including patient identification, to account for the lack of independence of observations.

	*S. haematobium* Mauritania	*S. haematobium* Senegal
*S. haematobium* Mali	-	Rmax (*p* = 0.002), EP (*p* = 0.006) EL (*p* = 0.003), ER (*p* = 0.006) BE20 (*p* = 0.0007), BE35 (*p* = 0.001) BE50 (*p* = 0.010), LWR (*p* = 0.0004) ERatio (*p* = 0.019), ESR (*p* = 0.0006)
*S. haematobium* Mauritania		-

**Table 5:** Mahalanobis distances between the three *S. haematobium* populations (Mali, Mauritania and Senegal) for the two discriminant analyses performed: (**a**) all measurements (17) and (**b**) spine measurements (4).

**(a)**	**Mali**	**Mauritania**	**Senegal**
Mali	0.00		
Mauritania	1.68 *	0.00	
Senegal	1.23 *	1.71 *	0.00
**(b)**	**Mali**	**Mauritania**	**Senegal**
Mali	0.00		
Mauritania	0.59 *	0.00	
Senegal	0.64 *	0.62	0.00

* Statistically different over 1000 permutations using the Bonferroni correction (*p* < 0.05).

**Table 6:** Number and percentage (%) of *S. haematobium* eggs detected in each morphotype (round, elongated and spindle) from Mali, Mauritania and Senegal.

	Round	Elongated	Spindle
Mali (N = 68)	26 (39.4%)	28 (42.4%)	12 (18.2%)
Mauritania (N = 55)	19 (34.5%)	20 (36.4%)	16 (29.1%)
Senegal (N = 41)	10 (25.0%)	14 (34.1%)	17 (41.5%)

**Table 7:** Mahalanobis distances between the nine *S. haematobium* egg populations according to their morphotype (round, elongated and spindle) and geographical origin (Mali, Mauritania and Senegal) for the discriminant analysis performed: (**a**) all measurements analyzed and (**b**) spine measurements analyzed.

**(a)**		**Mali**	**Mauritania**	**Senegal**
ROUND	Mali	0.00		
	Mauritania	3.19 *	0.00	
	Senegal	2.68 *	3.43 *	0.00
ELONGATED	Mali	0.00		
	Mauritania	2.35 *	0.00	
	Senegal	1.71	2.50 *	0.00
SPINDLE	Mali	0.00		
	Mauritania	2.67	0.00	
	Senegal	2.09	3.62 *	0.00
**(b)**		**Mali**	**Mauritania**	**Senegal**
ROUND	Mali	0.00		
	Mauritania	0.89	0.00	
	Senegal	1.29*	1.02	0.00
ELONGATED	Mali	0.00		
	Mauritania	0.82	0.00	
	Senegal	0.99	1.03	0.00
SPINDLE	Mali	0.00		
	Mauritania	0.69	0.00	
	Senegal	1.10	0.79	0.00

* Statistically different over 1000 permutations using the Bonferroni correction (*p* < 0.05).

## Data Availability

Not applicable.
